# Acupoint catgut embedding exerts node-dependent effects on leptin resistance–related signaling and the ghrelin–GOAT pathway in obese mice

**DOI:** 10.3389/fnut.2026.1853669

**Published:** 2026-09-15

**Authors:** Qiwang Yang, Han Yang, Jinjiao Zhang, Nenggui Xu, Chuanwu Zhu, Wei Yi, Hanqing Huang

**Affiliations:** 1 Department of Rehabilitation Medicine, The Fifth Affiliated Hospital of Southern Medical University, Guangzhou, China; 2 Medical College of Acupuncture Moxibustion and Rehabilitation, Guangzhou University of Chinese Medicine, Guangzhou, China; 3 Guangdong Provincial Key Laboratory of Acupuncture and Moxibustion of Chinese Medicine, Guangzhou University of Chinese Medicine, Guangzhou, China; 4 The First Clinical Medical College, Guangzhou University of Chinese Medicine, Guangzhou, China; 5 School of Traditional Chinese Medicine, Southern Medical University, Guangzhou, China; 6 Department of Plastic and Aesthetic Surgery, The People’s Hospital of Baoan Shenzhen, The Second Affiliated Hospital of Shenzhen University, Shenzhen, Guangdong, China

**Keywords:** acupoint catgut embedding, ghrelin-GOAT pathway, hypothalamus, leptin resistance, obesity

## Abstract

**Background:**

Obesity is a major metabolic health problem with limited effective treatments. Acupoint catgut embedding (ACE) has shown therapeutic potential; however, its underlying mechanisms remain unclear. This study aimed to investigate the effects of ACE on leptin resistance and the Ghrelin-GOAT pathway in obese mice.

**Methods:**

Diet-induced obesity was established in C57BL/6 J mice. A total of 51 mice were included in the final analysis across six groups: normal control, obese model control, ST36 sham ACE, ST36 verum ACE, ST25 ACE, and ST25 + ST36 ACE. ACE was administered once weekly for 5 weeks. Body weight, Lee’s index, and daily food intake were recorded. Serum and cerebrospinal fluid (CSF) leptin levels were measured to assess peripheral and central leptin availability. Hypothalamic SOCS3 and PTP1B protein expression and POMC/NPY mRNA levels were evaluated. Ghrelin-related indicators in gastric tissue and hypothalamus were also assessed.

**Results:**

At week 1, body weight and Lee’s index were lower in the ST36 verum ACE group than in the ST25 + ST36 group (*p* < 0.01), and the difference in Lee’s index remained significant at week 2 (*p* = 0.002). Food intake decreased transiently at week 1 in all obese groups, with a significant time effect (*p* < 0.001) but no group or group-by-time interaction effects (*p* > 0.05). For leptin resistance–related parameters, serum leptin levels in the model control group were significantly higher than those in the normal control group (*p* < 0.001), whereas CSF leptin levels were increased in the verum ST36 ACE group compared with the model control group (*p* < 0.001), accompanied by downregulation of hypothalamic SOCS3 and PTP1B expression (both *p* < 0.01). For downstream neuropeptides, POMC expression was upregulated and NPY expression was downregulated in the verum ST36 ACE group (both *p* < 0.001), with effects superior to those in the model control group and the ST25 ACE group (*p* < 0.05). In addition, within the ghrelin–GOAT pathway, gastric ghrelin and GOAT levels as well as hypothalamic GHSR-1a were higher in the verum ST36 ACE group than in the model control group (*p* < 0.001) and also greater than those in the ST25 ACE group (*p* < 0.05).

**Conclusion:**

ACE may increase central leptin availability, downregulate SOCS3 and PTP1B, regulate hypothalamic POMC/NPY expression, and modulate the ghrelin–GOAT pathway, supporting a node-dependent, multi-target mechanism underlying its anti-obesity effects.

## 1 Introduction

Obesity is a chronic metabolic disorder characterized by excessive fat accumulation and sustained imbalance between energy intake and expenditure, and its prevalence continues to rise worldwide. According to the World Health Organization (WHO), over 890 million adults were living with obesity in 2022, and obesity is a major risk factor for cardiovascular diseases, which remain a leading cause of death globally (1). Although lifestyle modification (diet and physical activity), pharmacological therapy, and bariatric surgery can induce short-term weight loss, long-term efficacy remains limited, largely due to poor adherence, adverse effects, and high rates of weight regain (2–5). These challenges highlight the need for alternative or complementary therapeutic approaches that are both effective and sustainable.

Acupoint catgut embedding (ACE) is a modified acupuncture technique in which an absorbable surgical thread is implanted into acupoints using a specialized needle. The thread is biodegradable, gradually absorbs over time, and provides continuous stimulation at the acupoints. Clinically, ACE has been widely applied in the management of obesity and has demonstrated consistent benefits in reducing body weight, suppressing appetite, and improving metabolic parameters (6, 7). Accumulating clinical and experimental evidence suggests that the therapeutic effects of ACE extend beyond simple caloric restriction and may involve regulation of neuroendocrine and metabolic pathways (8, 9). However, despite its established clinical efficacy, the biological mechanisms underlying the anti-obesity effects of ACE remain incompletely understood.

Previous mechanistic studies have indicated that ACE may influence appetite-related neuropeptides, leptin sensitivity, gastrointestinal hormones such as ghrelin, energy-sensing pathways including AMPK, inflammatory responses, and gut microbiota composition (10–12). Recent pharmacological evidence further indicates that activation of hypothalamic melanocortin-4 receptor signaling can regulate appetite and restore metabolic homeostasis in experimental hypothalamic obesity (13). While these findings suggest a multi-target regulatory profile, most studies have focused on isolated indicators or single pathways, and few have examined how central appetite-related hormonal systems are coordinated under ACE intervention. In particular, the mechanisms by which ACE may regulate key hormonal signals governing appetite and energy homeostasis have not been systematically clarified.

Among the hormonal systems implicated in obesity, leptin resistance and abnormal ghrelin signaling represent two central and complementary contributors to impaired appetite control. Leptin resistance is characterized by diminished central responsiveness despite elevated circulating leptin levels and is closely associated with impaired leptin transport across the blood–brain barrier (BBB) and enhanced negative regulation of hypothalamic leptin signaling mediated by suppressor of cytokine signaling 3 (SOCS3) and protein tyrosine phosphatase 1B (PTP1B) (14, 15). In parallel, the ghrelin–GOAT axis constitutes the best-characterized peripheral orexigenic hormonal system, and obesity is associated with disrupted ghrelin secretion patterns, reduced acylated ghrelin availability, and attenuated receptor responsiveness (16, 17). Increasing evidence suggests that these two systems interact functionally in the regulation of appetite and energy balance (18), making them particularly relevant targets for mechanistic investigation.

Based on these considerations, the present study employed a diet-induced obese mouse model to investigate whether ACE modulates appetite-related hormonal regulation through effects on leptin resistance and the ghrelin–GOAT pathway. By focusing on serum and CSF leptin levels, hypothalamic SOCS3 and PTP1B expression, and key components of the ghrelin–GOAT axis, this study aims to clarify the mechanistic basis underlying the anti-obesity effects of ACE and to provide experimental evidence linking its clinical efficacy to defined biological pathways.

## 2 Materials and methods

### 2.1 Animals

Male C57BL/6 J mice were selected based on established evidence demonstrating their susceptibility to high-fat diet–induced obesity and associated metabolic abnormalities (19). The total sample size was determined *a priori* based on previously published evidence (20) and power analysis (*α* = 0.05, power = 0.80), with allowance for potential animal loss and incomplete model induction during the high-fat diet–feeding period. Accordingly, 70 specific pathogen–free (SPF) male C57BL/6 J mice (4 weeks old) were purchased from Guangdong Zhiyuan Biotechnology Co., Ltd. (Guangdong, China). The animals were housed under controlled conditions with a temperature of 22 ± 2 °C, relative humidity of 50 ± 10%, and a 12-h light/dark cycle, with free access to food and water. After a 1-week acclimation period, 6 mice were selected and maintained on a standard chow diet (Jiangsu Synergy Pharmaceutical & Bioengineering Co., Ltd., Jiangsu, China; energy composition: 12.7% fat, 23.0% protein, and 64.3% carbohydrates) and served as the normal control group. The remaining 64 mice were fed a high-fat diet (Jiangsu Synergy Pharmaceutical & Bioengineering Co., Ltd., Jiangsu, China; energy composition: 60.65% fat, 18.14% protein, and 21.22% carbohydrates) for 12 weeks. Successful establishment of the obesity model was defined as a body weight at least 20% higher than the mean body weight of the normal control group after 12 weeks of feeding.

All animal experiments were approved by the Animal Ethics Committee of Guangzhou University of Chinese Medicine (Approval No. 20230329003) and were conducted in accordance with the ARRIVE guidelines (version 2.0) and the principles of replacement, reduction, and refinement (3Rs) at the South China Research Institute of Acupuncture and Moxibustion between April 27, 2023, and November 7, 2023. Animals were monitored daily throughout the study for general health status and signs of distress; no predefined humane endpoints were triggered. An overview of the experimental workflow and animal grouping is shown in Figure 1.

**Figure 1:**
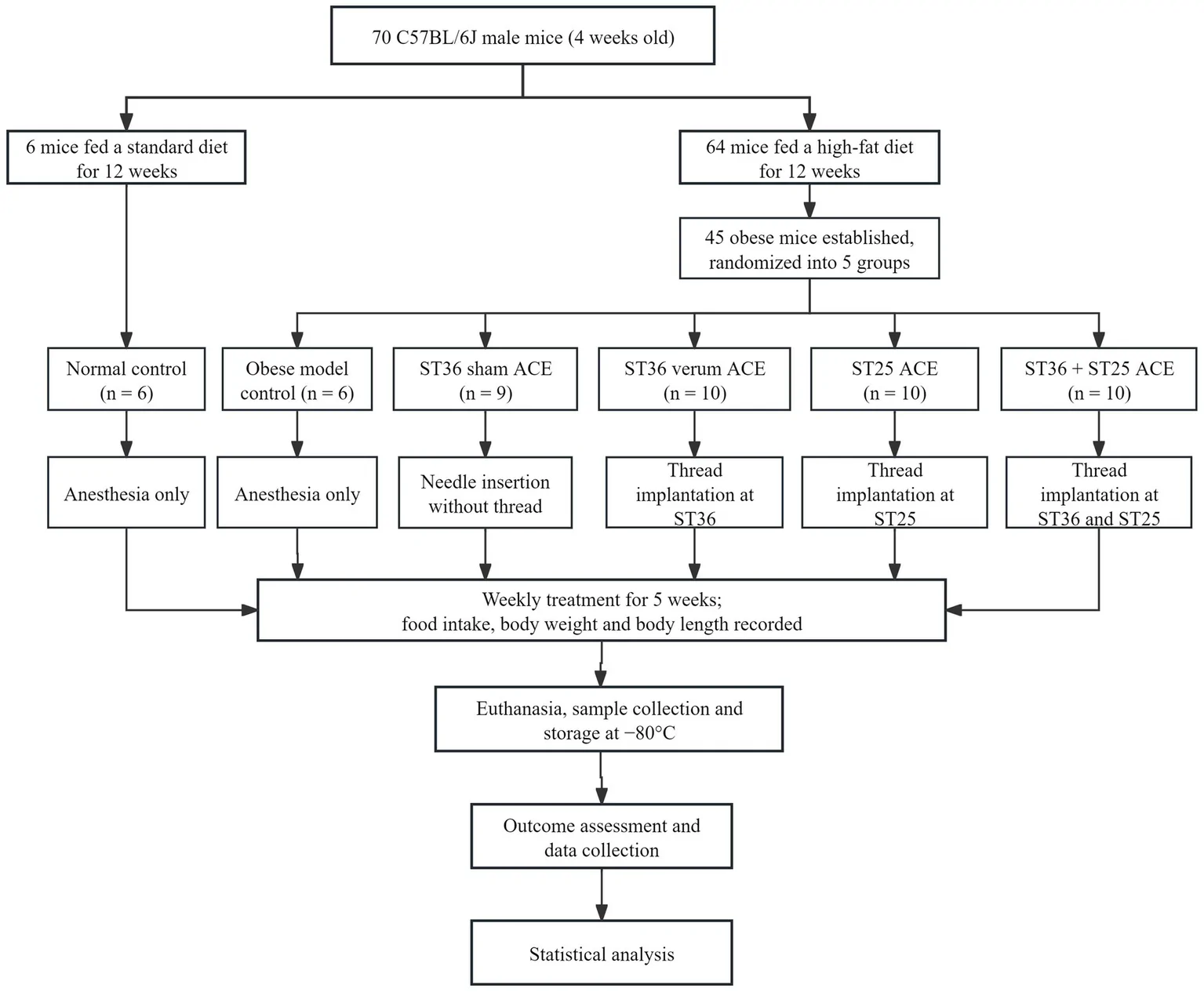
Experimental design and animal grouping.

### 2.2 Allocation and grouping

After completion of high-fat diet feeding, 45 mice met the predefined criteria for diet-induced obesity and were included in subsequent allocation. To establish an obese baseline reference while adhering to the 3Rs principles, a minimal number of obese mice (*n* = 6) was assigned to the obese model control group, which received no intervention and served as the disease baseline. The remaining 39 obese mice were allocated to four intervention groups, with slightly larger prespecified sample sizes for the verum ACE groups. Accordingly, the 45 diet-induced obese mice were allocated to the final groups as follows: obese model control group (*n* = 6), ST36 (Zusanli) sham ACE group (*n* = 9), ST36 (Zusanli) verum ACE group (*n* = 10), ST25 (Tianshu) ACE group (*n* = 10), and ST25 plus ST36 ACE group (*n* = 10). Randomization of the obese mice was performed using a computer-generated sequence to minimize allocation bias. Together with the normal control group which was maintained on a standard chow diet without any intervention and served as the healthy baseline (*n* = 6), the experiment comprised six groups in total.

### 2.3 Intervention procedures

At the start of the intervention, all mice were switched to a standard chow diet, which was maintained throughout the intervention period. Based on previous studies and experimental evidence (21), ST36 (Zusanli) and ST25 (Tianshu), two acupoints closely associated with obesity, were selected for intervention. ST36 is located in the muscle 5 mm below the head of the fibula at the knee joint, and ST25 is located approximately 5 mm lateral to the umbilicus.

All ACE procedures were performed under isoflurane anesthesia (induction: 3–5%; maintenance: 1.5–2% in oxygen). For the verum ACE groups (ST36, ST25, and ST36 + ST25), a 4 mm segment of absorbable surgical poly(glycolide-co-lactide) (PGLA) thread (Shandong Kanglida Medical Products Co., Ltd.) was cut and pre-loaded into a 27G needle (Zhejiang Beipu Medical Technology Co., Ltd.). The needle was inserted into the muscle layer at the corresponding acupoints, and the thread was delivered into the tissue by rotating the needle during withdrawal; the needle was then completely removed, leaving only the thread in place. The sham ACE group underwent the identical needle insertion procedure without thread implantation. Mice in the normal control and model control groups received anesthesia only. Interventions were administered once weekly for five consecutive weeks. During this period, all mice had free access to food and water, and body weight and body length were recorded weekly.

### 2.4 Sample collection

At the end of the intervention period, mice were fasted for 12 h with free access to water and anesthetized with pentobarbital sodium (75 mg/kg, intraperitoneally) (22). Under this level of anesthesia, cerebrospinal fluid (CSF) and blood samples were obtained. CSF was collected by puncture of the cisterna magna using a standard procedure. Mice were placed in a stereotaxic frame with the head flexed to expose the occipital region. Under a stereomicroscope, a glass capillary tube was carefully inserted into the cisterna magna, and clear, non-bloody CSF was allowed to flow spontaneously into the capillary. Blood samples were obtained immediately after CSF collection by cardiac puncture. Whole blood was allowed to clot at room temperature for 30 min and then centrifuged at 3000 rpm for 10 min to separate serum, which was then aliquoted.

Following blood collection, animals received an additional intraperitoneal injection of pentobarbital sodium to achieve a total dose ≥150 mg/kg for euthanasia, in accordance with the American Veterinary Medical Association (AVMA) Guidelines for the Euthanasia of Animals (2020). Death was confirmed by cessation of respiration and heartbeat prior to tissue collection. No additional physical euthanasia methods were applied. The hypothalamus was then rapidly dissected on ice and processed as described below. The hypothalamic region was isolated according to anatomical landmarks, including the area between the optic chiasm and mammillary bodies. The stomach was subsequently excised via a midline abdominal incision. After ligation of the cardia and pylorus, the stomach was removed, emptied, and rinsed with cold saline. Gastric mucosa from the fundus and corpus was gently scraped, weighed, and homogenized in ice-cold buffer. Aliquots of the homogenate were prepared for further analysis.

All collected samples, including CSF, serum, hypothalamic tissue, and gastric tissue, were immediately snap-frozen in liquid nitrogen and stored at −80 °C until subsequent analyses.

### 2.5 Outcome measurements

Outcome measurements comprised general obesity-related indices as well as biochemical and molecular markers associated with leptin- and ghrelin-related pathways.

#### 2.5.1 Body weight and Lee’s index

Body weight was measured weekly using an electronic balance with an accuracy of 0.01 g. Measurements were performed at a fixed time each week to minimize circadian variation. All animals were weighed by the same investigator under standardized conditions. Lee’s index was calculated to evaluate the degree of obesity based on body weight and body length (23). Body length (nose-to-anus distance) was measured under anesthesia using a vernier caliper. Lee’s index was calculated as:

$${\mathrm{Lee}}^{'}s\;\text{index}=\frac{\sqrt[{3}]{\text{body weight}\;(g)}\;}{\text{body length}\;(\mathrm{cm})}$$

$$\text{Linearformat}:{\mathrm{Lee}}^{'}\text{sindex}={\left[\text{bodyweight}(g)\right]}^{\left(1/3\right)}/\text{bodylength}(\mathrm{cm})$$

#### 2.5.2 Food intake

Food intake was measured weekly at the cage level throughout the 5-week intervention. At the beginning of each 7-day measurement period, a pre-weighed amount of standard chow was provided to each cage, and the remaining feed was weighed after 7 days. Total food intake for each cage was calculated as the difference between the amount of feed provided and the amount remaining and was recorded in grams (g). Because the mice were group-housed, food intake was measured for each cage rather than for individual mice.

#### 2.5.3 ELISA analysis

Enzyme-linked immunosorbent assay (ELISA) was used to quantify serum and CSF leptin levels, gastric tissue and CSF ghrelin levels, gastric tissue ghrelin O-acyltransferase (GOAT) levels, and hypothalamic growth hormone secretagogue receptor 1a (GHSR-1a) levels. All measurements were performed using commercially available mouse ELISA kits (Jiangsu Meimian Industrial Co., Ltd., China; catalog numbers: MM-0622 M1 for leptin, MM-0621 M1 for ghrelin, MM-47126 M1 for GOAT, and MM-47229 M1 for GHSR-1a) according to the manufacturers’ instructions. Briefly, samples and standards were added to antibody-precoated plates and incubated with horseradish peroxidase–conjugated detection antibodies. After washing, substrate solution was added, and absorbance was measured at 450 nm using a microplate reader. Protein concentrations were calculated from standard curves generated for each assay.

#### 2.5.4 Western blot analysis

Hypothalamic tissues were homogenized in RIPA lysis buffer (Solarbio, China) supplemented with protease inhibitors. Total protein concentrations were determined using a BCA protein assay kit (Beyotime, China). Equal amounts of protein (approximately 80 μg per sample) were separated by SDS–polyacrylamide gel electrophoresis and transferred onto polyvinylidene fluoride (PVDF) membranes. After blocking with 5% non-fat milk for 1 h at room temperature, membranes were incubated overnight at 4 °C with primary antibodies against SOCS3 (1:500; Affinity Biosciences, China; Cat# DF6133) and PTP1B (1:500; Affinity Biosciences, China; Cat# AF6205). GAPDH (1:2000; Youkang, China; Cat# UM4002) was used as the loading control. After washing, membranes were incubated with HRP-conjugated secondary antibodies (1:4000; Affinity Biosciences, China) for 1 h at room temperature. Immunoreactive bands were visualized using an enhanced chemiluminescence (ECL) detection system, and band intensities were quantified by densitometry and normalized to GAPDH.

#### 2.5.5 RT-qPCR analysis

Total RNA was extracted from hypothalamic tissues with a commercially available RNA extraction kit and reverse-transcribed into complementary DNA (cDNA) according to the manufacturers’ instructions. Quantitative real-time PCR was performed with SYBR Green chemistry on an ABI 7500 real-time PCR system to assess the mRNA expression levels of proopiomelanocortin (POMC) and neuropeptide Y (NPY). Glyceraldehyde-3-phosphate dehydrogenase (GAPDH) was used as an internal reference gene. Across the 51 biological samples, the mean GAPDH Ct value was 14.54 ± 0.56 cycles after averaging the three technical replicates for each sample. The technical replicates showed good consistency, and melting-curve analysis showed a single predominant peak. Relative gene expression levels were calculated using the 2^−^ΔΔCt method. Primer sequences used for RT-qPCR are provided in Supplementary Table S1.

### 2.6 Statistical analysis

Statistical analyses were performed using IBM SPSS Statistics software (version 25.0). Continuous data are presented as mean ± standard deviation (SD) for normally distributed variables and as median (P25–P75) for non-normally distributed variables. Repeated-measures outcomes (body weight and Lee’s index) were analyzed using generalized estimating equations (GEE). Food-intake changes from the composition-weighted baseline proxy (g/mouse/week) were analyzed at the cage level using a linear mixed-effects model, with treatment cage as the experimental unit, week as the repeated measure, group, time, and group × time as fixed effects, and a compound-symmetry covariance structure estimated by restricted maximum likelihood. Single-measure outcomes were analyzed using one-way ANOVA or the Kruskal–Wallis H test, as appropriate. All statistical tests were two-tailed, with statistical significance set at *p* < 0.05, and sequential Bonferroni adjustment was applied for between-group pairwise comparisons.

For repeated-measures outcomes, week 1, week 2, and week 5 were predefined as the primary time points for statistical inference, whereas other time points were used mainly for trend visualization. Within-group comparisons focused on changes from week 0 to week 1 and from week 1 to week 5. Between-group pairwise comparisons were anchored to the ST36 verum ACE group as the primary reference and conducted according to a predefined hierarchy (model control vs. intervention groups; ST36 verum vs. sham; and ST36 verum vs. other acupoint protocols).

## 3 Results

### 3.1 Changes in body weight and Lee’s index

Body weight changes were analyzed using generalized estimating equations (GEE). Significant main effects of group and time, as well as a significant group-by-time interaction, were observed (*p* < 0.001 for all effects). Except for the normal control group, body weight decreased significantly during the first week across all experimental groups (*p* < 0.001) and remained relatively stable thereafter. At week 1, body weight in the ST36 verum group was significantly lower than that in the ST25 plus ST36 group (mean difference = −4.73 g, *p* = 0.001), whereas none of the between-group comparisons involving the ST36 verum group at week 2 or week 5 reached statistical significance (*p* > 0.05; Figure 2A).

**Figure 2:**
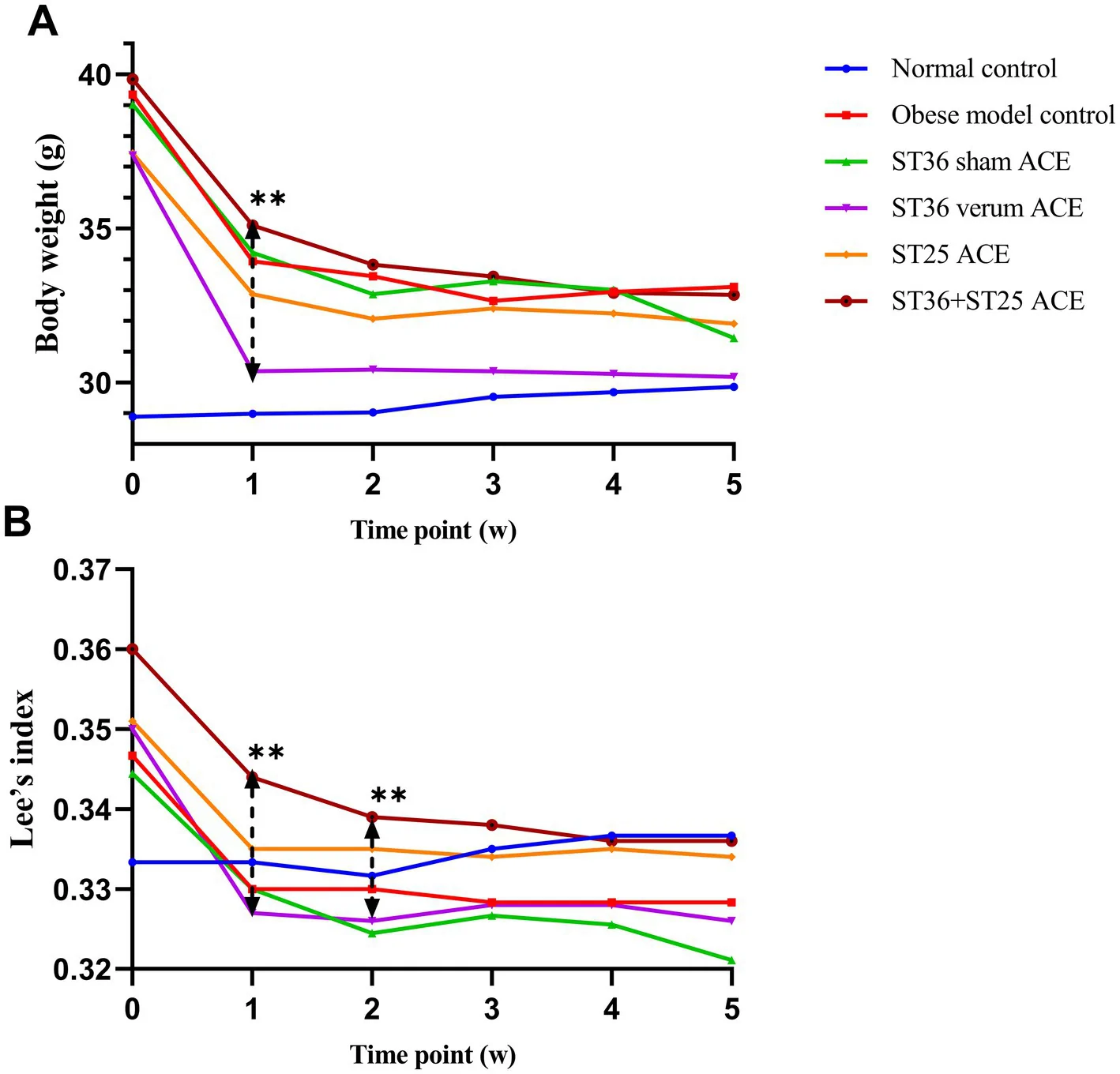
Body weight and Lee’s index changes over time **(A)** Body weight changes over time in different experimental groups. **(B)** Lee’s index changes over time in different experimental groups. **Indicates a significant difference between the ST36 verum group and the ST25 + ST36 group (*p* < 0.01).

Similar patterns were observed for Lee’s index. GEE analysis revealed significant main effects of group and time, as well as a significant group-by-time interaction (*p* < 0.001 for all effects). With the exception of the normal control group, Lee’s index decreased significantly during the first week across the experimental groups (*p* < 0.001) and showed no further significant changes thereafter. At week 1, Lee’s index in the ST36 verum group was significantly lower than that in the ST25 plus ST36 group (mean difference = −0.0167, *p* < 0.001), and this difference remained significant at week 2 (mean difference = −0.0122, *p* = 0.002), whereas no significant between-group differences were observed by week 5 (*p* > 0.05; Figure 2B).

### 3.2 Changes in weekly food intake

Food-intake changes relative to the composition-weighted baseline proxy are shown in Figure 3. The cage-level mean change in food intake remained close to zero in the normal control group during week 1, whereas marked decreases were observed in the obese model control and intervention groups, followed by varying degrees of recovery over the subsequent weeks. The linear mixed-effects model showed a significant main effect of time (*F* = 19.687, *p* < 0.001), whereas neither the main effect of group (*F* = 1.160, *p* = 0.392) nor the group-by-time interaction (*F* = 1.646, *p* = 0.089) was significant. These findings indicate that food intake changed over time, but that the overall magnitude of change and temporal pattern did not differ significantly among the six groups (Supplementary Tables S8–S9).

**Figure 3:**
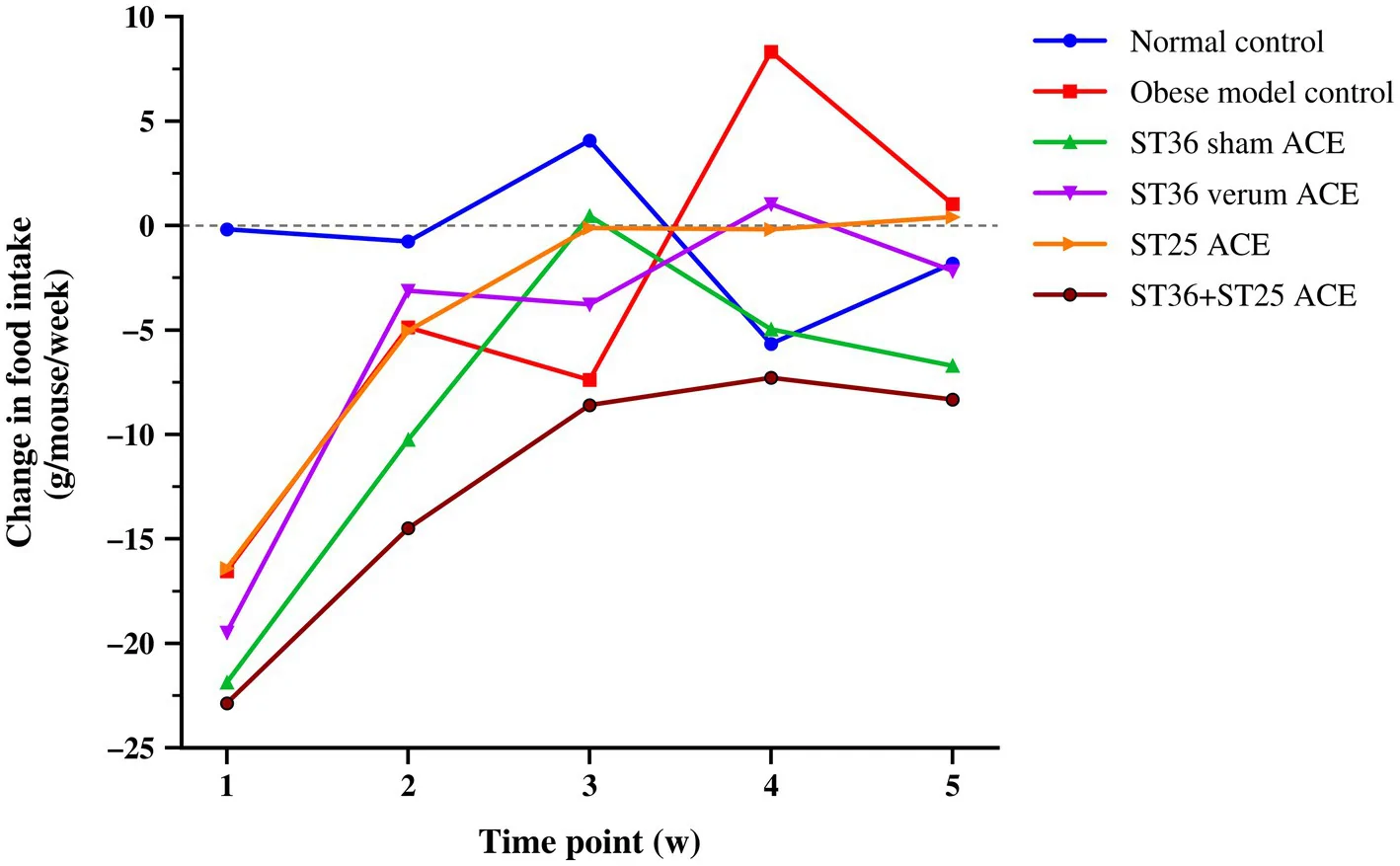
Changes in weekly food intake relative to the baseline proxy. Food intake was measured weekly at the cage level and expressed as g/mouse/week. Lines represent group mean changes from the composition-weighted pre-intervention baseline proxy at weeks 1–5; the horizontal dashed line represents the baseline reference (change = 0).

### 3.3 Serum and CSF leptin levels

Serum leptin levels differed significantly among groups, as determined by the Kruskal–Wallis H test (H = 41.18, *p* < 0.001). *Post hoc* analyses showed that serum leptin levels were significantly higher in the model control group than in the normal control group (*p* < 0.001), whereas no significant differences were observed among the obese groups (all *p* > 0.05).

CSF leptin levels also showed a significant overall group effect based on one-way ANOVA (*p* < 0.001). *Post hoc* comparisons indicated that leptin levels were significantly higher in the ST36 verum group than in the model control group (*p* < 0.001). No significant differences were detected in the predefined comparisons: normal control versus model control, ST36 verum versus ST36 sham, ST36 verum versus ST25, or ST36 verum versus ST25 plus ST36 (all *p* > 0.05; Figure 4A).

**Figure 4:**
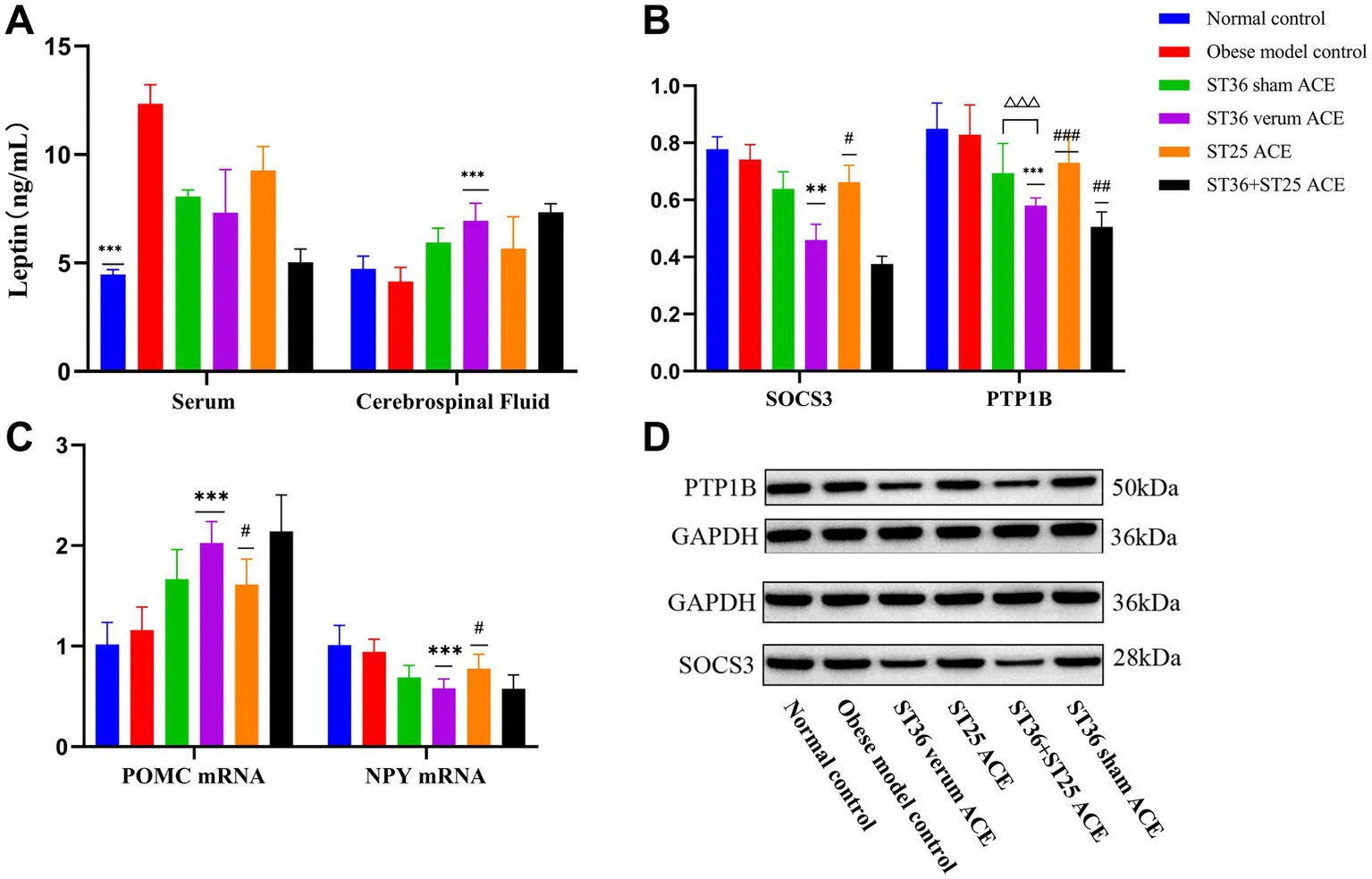
Regulation of leptin resistance-related mechanisms by ACE in obese mice. **(A)** Serum and cerebrospinal fluid leptin levels. **(B)** Protein expression of hypothalamic SOCS3 and PTP1B. **(C)** Relative mRNA expression of hypothalamic POMC and NPY. **(D)** Western blot images showing SOCS3 and PTP1B expression. GAPDH was used as a loading control.\rStatistical significance: Symbol * indicates comparison between the obese model control and experimental groups; △ indicates comparison between ST36 verum ACE and sham ACE groups; # indicates comparison between ST36 verum ACE and ST25 or ST36 + ST25 ACE groups. * / △ / # *p* < 0.05; ** / △△ / ## *p* < 0.01; *** / △△△ / ### *p* < 0.001.

### 3.4 Hypothalamic leptin resistance–related signaling

Hypothalamic SOCS3 protein expression differed significantly among groups, as assessed by the Kruskal–Wallis H test (H = 39.97, *p* < 0.001). Post hoc analyses showed that SOCS3 expression was significantly lower in the ST36 verum group than in the model control group (*p* < 0.001) and the ST25 group (*p* < 0.05). No significant differences were observed for the following comparisons: normal control versus model control, ST36 verum versus ST36 sham, or ST36 verum versus ST25 plus ST36 (all *p* > 0.05).

PTP1B protein expression in the hypothalamus also showed significant between-group differences based on one-way ANOVA (*p* < 0.001). Compared with the model control group, PTP1B expression was significantly reduced in the ST36 verum group (*p* < 0.001) and was also lower than that in the ST36 sham group (*p* < 0.001), whereas no significant difference was observed between the normal control and model control groups (*p* > 0.05). Among different acupoint protocols, PTP1B expression was lower in the ST25 group than in the ST36 verum group (*p* < 0.001), while the ST36 verum group showed lower PTP1B levels than the ST25 plus ST36 group (*p* < 0.01; Figure 4B). Representative Western blot images are shown in Figure 4D.

Hypothalamic POMC mRNA expression differed significantly among groups based on one-way ANOVA (*p* < 0.001). *Post hoc* analyses showed that POMC mRNA levels were significantly higher in the ST36 verum group than in the model control group (*p* < 0.001), whereas no significant differences were observed between the normal control and model control groups or between the ST36 sham and model control groups (*p* > 0.05). Among different acupoint protocols, POMC mRNA expression was lower in the ST25 group than in the ST36 verum group (*p* < 0.05), while no significant difference was detected between the ST36 verum and ST25 plus ST36 groups (*p* > 0.05).

Significant between-group differences were also observed in hypothalamic NPY mRNA expression (one-way ANOVA, *p* < 0.001). Compared with the model control group, NPY mRNA levels were significantly reduced in the ST36 verum group (*p* < 0.001), whereas no significant differences were detected between the normal control and model control groups or between the ST36 sham and model control groups (p > 0.05). Among acupoint protocols, NPY mRNA expression was higher in the ST25 group than in the ST36 verum group (*p* < 0.05), while no significant difference was observed between the ST36 verum and ST25 plus ST36 groups (*p* > 0.05; Figure 4C).

### 3.5 Ghrelin–GOAT pathway–related molecular outcomes

Gastric ghrelin levels differed significantly among groups, as determined by one-way ANOVA (*p* < 0.001). *Post hoc* analyses showed that gastric ghrelin levels were significantly lower in the model control group than in the normal control group (*p* < 0.001) and the ST36 verum group (p < 0.001). Compared with the ST36 verum group, gastric ghrelin levels were also significantly reduced in the ST36 sham group (*p* < 0.05). Among different acupoint protocols, the ST25 group exhibited significantly lower gastric ghrelin levels than the ST36 verum group (*p* < 0.001), whereas no significant difference was observed between the ST36 verum and ST25 plus ST36 groups (*p* > 0.05). CSF ghrelin levels showed significant between-group differences according to the Kruskal–Wallis H test (*p* < 0.001). Compared with the normal control group, ghrelin levels were markedly reduced in the model control group (*p* < 0.001) and were also lower than those in the ST36 verum group (*p* < 0.01). No significant differences were detected for the following comparisons: ST36 sham versus ST36 verum, ST36 verum versus ST25, or ST36 verum versus ST25 plus ST36 (all *p* > 0.05; Figure 5A).

**Figure 5:**
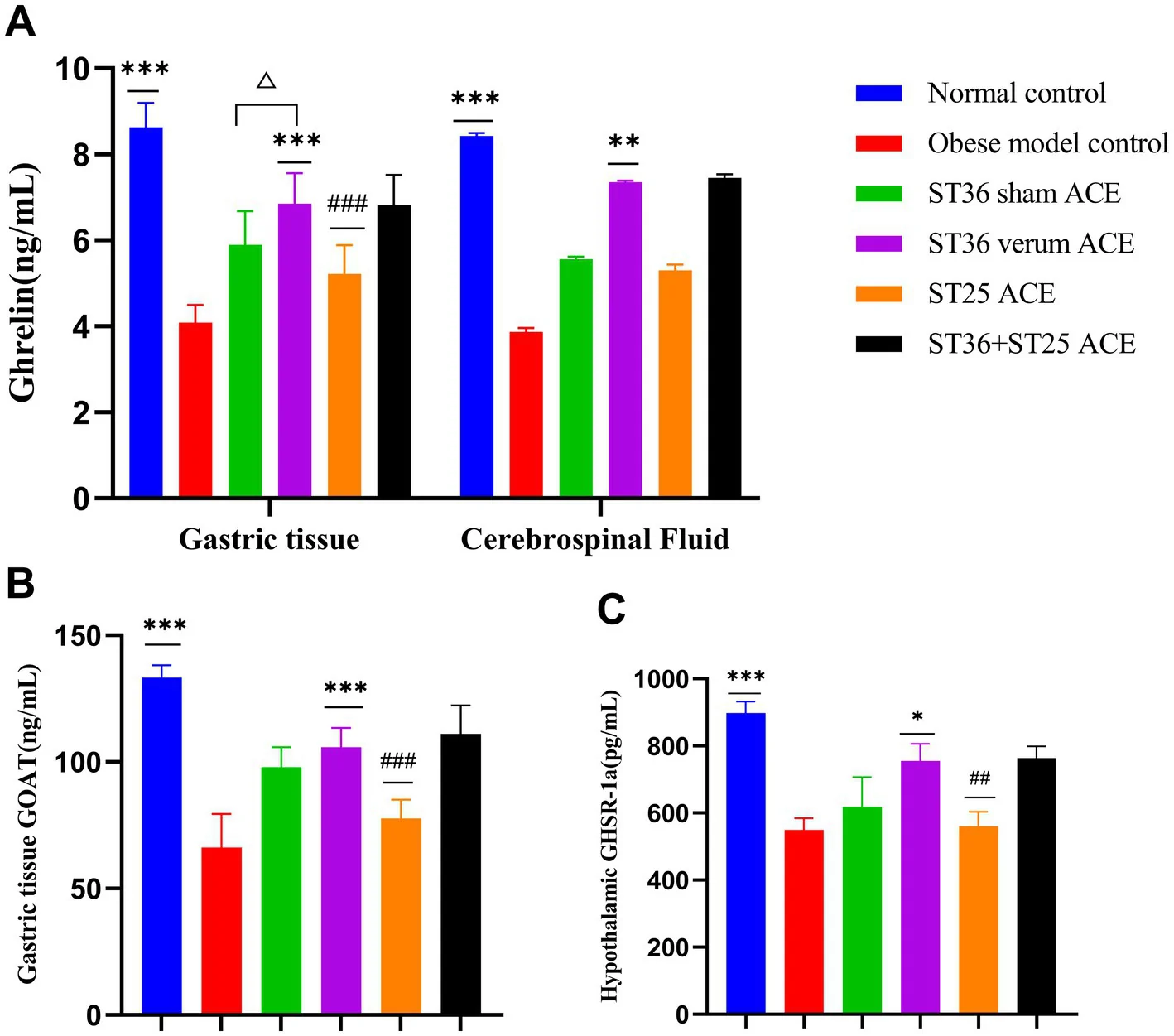
Effects of ACE on the ghrelin-GOAT signaling pathway in obese mice. **(A)** Ghrelin levels in gastric tissue and cerebrospinal fluid. **(B)** GOAT levels in gastric tissue. **(C)** Hypothalamic GHSR-1a levels. Statistical significance: Symbol * indicates comparison between the obese model control and experimental groups; △ indicates comparison between ST36 verum ACE and sham ACE groups; # indicates comparison between ST36 verum ACE and ST25 or ST36 + ST25 ACE groups. * / △ / # *p* < 0.05; ** / △△ / ## p < 0.01; *** / △△△ / ### *p* < 0.001.

Significant differences in gastric GOAT levels were observed among groups based on one-way ANOVA (*p* < 0.001). Gastric GOAT levels in the model control group were significantly lower than those in the normal control group (*p* < 0.001) and the ST36 verum group (*p* < 0.001). No significant difference was observed between the ST36 sham and ST36 verum groups (*p* > 0.05). Among acupoint protocols, gastric GOAT levels were significantly reduced in the ST25 group compared with the ST36 verum group (*p* < 0.001), whereas no significant difference was detected between the ST36 verum and ST25 plus ST36 groups (*p* > 0.05; Figure 5B).

Hypothalamic GHSR-1a protein expression differed significantly among groups according to the Kruskal–Wallis H test (*p* < 0.001). Compared with the normal control group, GHSR-1a levels were significantly reduced in the model control group (*p* < 0.001) and were also lower than those in the ST36 verum group (*p* < 0.05). Further comparisons among acupoint protocols showed that hypothalamic GHSR-1a expression was significantly lower in the ST25 group than in the ST36 verum group (*p* < 0.01), whereas no significant difference was observed between the ST36 verum and ST25 plus ST36 groups (*p* > 0.05; Figure 5C).

## 4 Discussion

In the present study, body weight and Lee’s index were used as general phenotypic indicators and exhibited broadly parallel trajectories during the intervention period. Except for the normal control group, all obese groups showed significant reductions at week 1, followed by stabilization from week 2 to week 5. Similar early-phase decreases have been reported in previous experimental studies of ACE during the initial stage of intervention (24, 25). However, this early decline should not necessarily be attributed solely to ACE. Consistent with the phenotypic changes, food intake remained close to baseline in the normal control group during week 1 but decreased markedly in both the obese model control and intervention groups. Previous research has also shown that changes in dietary composition can rapidly alter food intake in C57BL/6 mice (26). Therefore, the reductions in body weight and Lee’s index observed at week 1 may have been related to reduced food consumption following the switch from the high-fat diet to standard chow, rather than representing a treatment-specific effect of ACE alone. The subsequent plateau from week 2 to week 5 suggests that, under the present experimental conditions, ACE did not exert a sustained weight-reduction effect. Between-group comparisons revealed that at week 1, verum ST36 stimulation resulted in lower body weight and Lee’s index compared with the ST25 plus ST36 combination group, with the difference in Lee’s index persisting at week 2. This observation contrasts with the commonly presumed synergistic effects of multi-acupoint stimulation reported in some studies (10). ST36 is widely recognized as a key acupoint in metabolic regulation and energy homeostasis (27). However, combined stimulation with ST25 did not yield a clear synergistic effect. This finding may reflect non-additive or interfering neuroendocrine interactions between simultaneous stimulation at ST36 and ST25.

Under normal metabolic conditions, circulating leptin crosses the BBB via a receptor-mediated transport system to enter the CSF and exert central anorexigenic effects (28, 29). In obesity, this process is frequently disrupted, resulting in elevated peripheral leptin levels without a corresponding increase in CSF leptin, leading to insufficient central leptin signaling—a phenomenon termed leptin resistance, which is attributed in part to impaired leptin transport across the BBB (30, 31). Consistent with this paradigm, the present study showed that serum leptin levels were significantly elevated in obese model mice, whereas CSF leptin levels did not differ from those of normal controls, indicating a mismatch between peripheral and central leptin levels. When ACE intervention was applied, CSF leptin levels were higher in the ST36 verum group than in the obese model control group, suggesting increased central leptin availability. Although this increase may be compatible with enhanced leptin entry into the central nervous system, CSF leptin concentrations are also affected by clearance. Therefore, this finding alone is insufficient to establish enhanced leptin transport across the BBB. Similar improvements in leptin-related central signaling following acupuncture- or ACE-based interventions have been reported in obese animal models, potentially through modulation of hypothalamic leptin-related pathways (10, 32). However, when comparisons were restricted to the predefined intervention groups, no significant differences in CSF leptin levels were observed between the ST36 verum group and the ST36 sham, ST25, or ST25 plus ST36 groups. This finding indicates that the observed increase in CSF leptin was not strongly dependent on acupoint specificity or combination strategy, suggesting instead that ACE may exert a more generalized effect on central leptin-related regulation.

Leptin resistance is a multifactorial pathological process involving not only impaired transport across the BBB but also negative regulation of downstream signaling and dysregulation of appetite-related neuropeptides (10, 33, 34). In line with this, the present study showed that SOCS3, PTP1B, POMC, and NPY in the obese model group remained comparable to those in the normal control group, further reflecting the blunted central leptin signaling characteristic of leptin resistance. ACE intervention was associated with reduced SOCS3 expression in the ST36 verum group compared with the model control and ST25 groups, whereas no significant difference was observed between the verum and sham groups, suggesting that SOCS3 suppression may reflect a non-specific metabolic or neuroendocrine response to embedding-related stimulation rather than a predominantly acupoint-dependent effect. Consistent with this interpretation, previous studies (35, 36) have shown that hypothalamic SOCS3 expression is readily modulated by non-specific metabolic interventions, such as physical exercise or systemic anti-inflammatory regulation, and may therefore represent a marker of global metabolic improvement rather than acupoint-specific signaling modulation. In contrast, PTP1B expression was markedly reduced in the ST36 verum group compared with both the model control and sham groups, indicating a more pronounced acupoint-specific effect at ST36. This finding is consistent with previous reports identifying PTP1B as a critical regulatory node in leptin resistance and a sensitive target for metabolic interventions (37, 38). Notably, although ST25 stimulation alone exerted a strong inhibitory effect on PTP1B expression, this effect was attenuated when combined with ST36, suggesting that acupoint combination does not necessarily produce synergistic regulation and may involve signal interference or overlapping feedback mechanisms. At the level of downstream neuropeptides, POMC mRNA expression was increased and NPY mRNA expression was decreased in the ST36 verum group compared with the model control group; however, differences between the verum and sham groups were inconsistent, indicating partial non-specificity in neuropeptide regulation. By comparison, the reduction of NPY expression showed greater sensitivity to acupoint selection, supporting its potential role as a functional downstream mediator of leptin resistance improvement (39, 40). These observations suggest that ACE modulates hypothalamic leptin resistance through multiple signaling targets, with PTP1B exhibiting the strongest acupoint specificity, NPY showing relative sensitivity to acupoint selection at the downstream level, whereas SOCS3 and POMC appear to be more influenced by non-specific embedding-related effects.

The ghrelin–GOAT pathway constitutes an important peripheral–central signaling axis involved in energy homeostasis (41–43). In the present study, obesity was associated with significantly reduced gastric ghrelin, GOAT, and hypothalamic GHSR-1a expression, indicating suppression of this pathway under high-fat feeding conditions, which is consistent with previous observations in obese animal models (44). ACE partially reversed these alterations, although the regulatory pattern differed across distinct nodes of the pathway. At the peripheral level, gastric ghrelin expression was significantly increased in the ST36 verum group compared with both the model control and sham groups, suggesting a site-specific effect of ST36 stimulation on ghrelin secretion. Similar changes of ghrelin-related signaling have been reported following exercise or hypoxic interventions, supporting the biological plausibility of this finding (45–47). In contrast, increases in CSF ghrelin, gastric GOAT, and hypothalamic GHSR-1a expression did not consistently differ between verum and sham embedding, indicating that regulation at downstream nodes of the pathway may predominantly reflect non-specific embedding-related stimulation rather than strict acupoint specificity. Moreover, comparisons among acupoint protocols revealed that ST25 stimulation was less effective than ST36 in modulating ghrelin–GOAT signaling, while combined acupoint intervention failed to produce additive or synergistic effects. Collectively, these findings suggest that ACE modulates the ghrelin–GOAT pathway in a node-dependent manner, with greater acupoint specificity at the peripheral initiation step and more generalized regulatory effects along downstream components, thereby contributing to metabolic normalization rather than direct stimulation of orexigenic drive.

Taken together, the present findings suggest that ACE may exert its anti-obesity effects through a multi-level and multi-target regulatory pattern rather than through a single dominant pathway. By concurrently modulating leptin resistance–related signaling and the ghrelin–GOAT axis, ACE may influence both central and peripheral components of metabolic regulation in a hierarchical and node-dependent manner. The heterogeneous response profiles observed across different molecular indicators and acupoint protocols further indicate that the effects of ACE are not simply additive, but instead reflect coordinated regulation across distinct signaling nodes. This integrated regulatory framework provides a coherent biological basis for the observed weight-related outcomes and is summarized in the proposed mechanistic model (Figure 6), which offers an overall conceptual view of the mechanisms underlying the anti-obesity actions of ACE.

**Figure 6:**
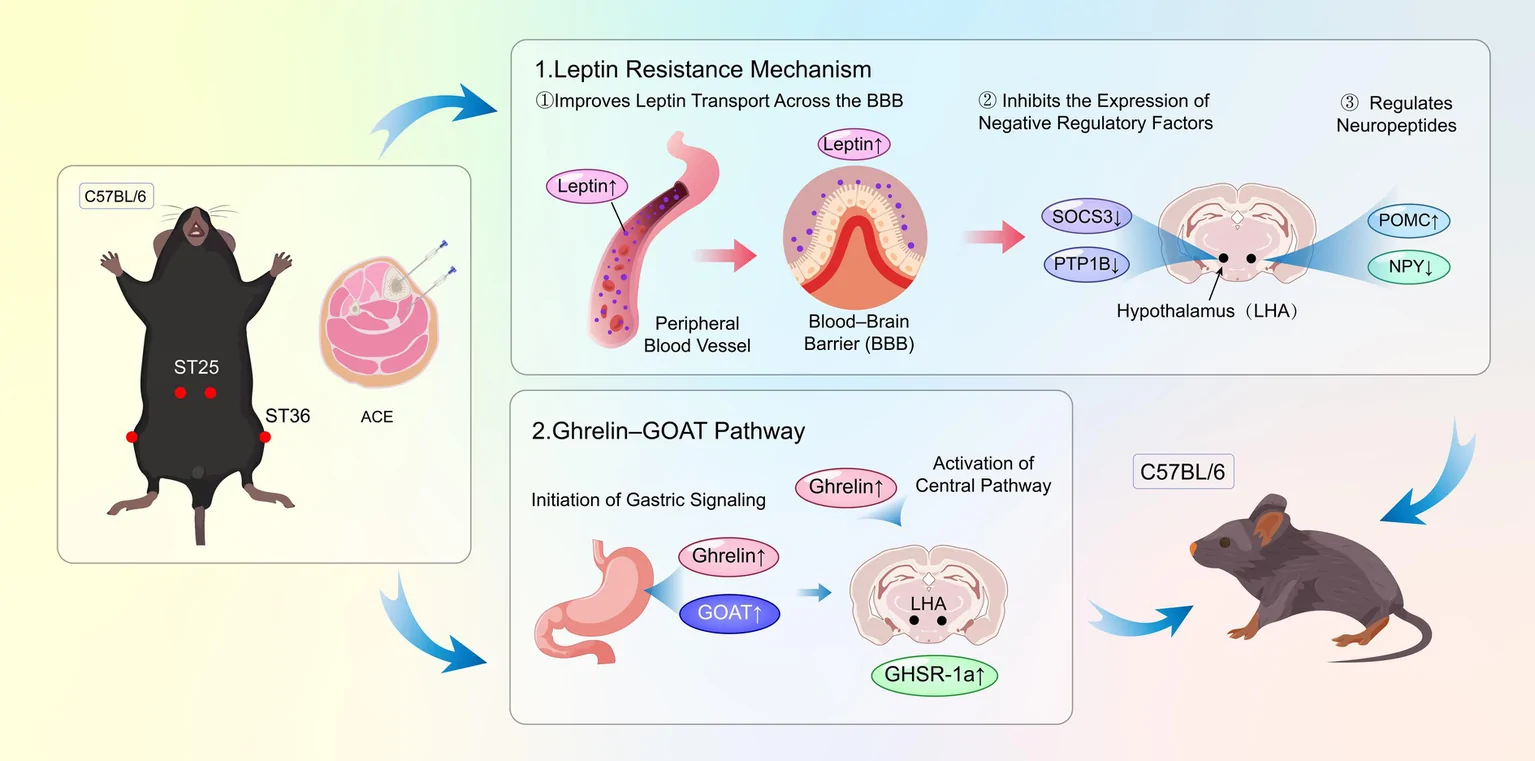
Schematic illustration of the proposed mechanisms underlying the effects of ACE in obese mice.

Several limitations of the present study should be acknowledged. First, the animal experiments were conducted with a relatively limited sample size and exclusively in male C57BL/6 J mice, and potential sex-related differences and the influence of genetic background were not examined. Second, the intervention period was relatively short, which may not fully capture long-term metabolic adaptations or neuroplastic changes induced by ACE. Third, the dietary switch from high-fat to chow diet at the start of the intervention, while it unified the dietary conditions across groups, introduced a confounding factor that makes it difficult to distinguish the specific effects of ACE from the effects of dietary change itself. In addition, the present analysis focused primarily on key hypothalamic and gastric signaling pathways, while other metabolically relevant tissues and systems, such as adipose tissue and the gut microbiota, were not systematically evaluated, which may lead to an underestimation of the broader integrative regulatory effects of ACE. Future studies incorporating larger sample sizes, longer observation periods, continued high-fat feeding, and multi-omics approaches will be important to further elucidate the integrated mechanisms of ACE.

## Data Availability

The raw data supporting the conclusions of this article will be made available by the authors, without undue reservation.
