# Supplementary material for: Acupoint catgut embedding exerts node-dependent effects on leptin resistance–related signaling and the ghrelin–GOAT pathway in obese mice
Source: Front Nutr. 2026 Sep 15;13:1853669. doi: 10.3389/fnut.2026.1853669 (PMC13631939; doi:10.3389/fnut.2026.1853669)
Supplement: Supplementary file 1 [file Supplementary_file_1.DOCX]

## Supplementary Table S1 Primer sequences used for RT-qPCR

| **Gene** | **Primer** | **Sequence (5′–3′)** | **Amplicon size (bp)** |
| --- | --- | --- | --- |
| POMC | F | CGCTGGAGACGCCCGTGTTTC | 193 |
|  | R | CGTGGACTCGGCTCTGGACTGC |  |
| NPY | F | GTGTGGACTGACCCTCGCTCTATC | 155 |
|  | R | TGGTGATGAGATTGATGTAGTGTCGC |  |
| GAPDH | F | CGGTGCTGAGTATGTCGTGGAGTC | 100 |
|  | R | GGCGGAGATGATGACCCTTTTG |  |

## Supplementary Tables S2–S4：GEE results for body weight

Table S2. GEE main effects for body weight

| **Source** | **Wald *χ²*** | **Degrees of Freedom** | ***P* value** |
| --- | --- | --- | --- |
| **Group** | 59.248435 | 5 | ＜0.001 |
| **Time Points** | 578.347806 | 5 | ＜0.001 |
| **Group * Time Points** | 1530.126106 | 25 | ＜0.001 |

Table S3. Within-group comparisons of body weight at different time points

| **Group** | **Time Point Comparison** | **Mean Difference** | **Standard Error** | **Sequential Bonferroni**  ***P* Value** |
| --- | --- | --- | --- | --- |
| Normal control  (*n*=6) | week0 vs week1 | -0.0933 | 0.17246 | 1 |
|  | week1 vs week5 | -0.88 | 0.3559 | 1 |
| Obese model control  (*n*=6) | week0 vs week1 | 10.3667 | 1.85628 | ＜0.001 |
|  | week1 vs week5 | 0.835 | 0.35826 | 1 |
| ST36 sham ACE  (*n*=9) | week0 vs week1 | 10.0406 | 1.24906 | ＜0.001 |
|  | week1 vs week5 | 2.7744 | 0.81265 | 0.273 |
| ST36 verum ACE  (*n*=10) | week0 vs week1 | 8.3903 | 0.83267 | ＜0.001 |
|  | week1 vs week5 | 0.174 | 0.60537 | 1 |
| ST25 ACE  (*n*=10) | week0 vs week1 | 8.4543 | 1.30552 | ＜0.001 |
|  | week1 vs week5 | 0.956 | 0.7872 | 1 |
| ST25+ ST36 ACE  (*n*=10) | week0 vs week1 | 10.8543 | 1.08016 | ＜0.001 |
|  | week1 vs week5 | 1.9917 | 1.42669 | 1 |

**Abbreviations:** ST36, Zusanli; ST25, Tianshu; ACE, acupoint catgut embedding

Table S4. Between-group comparisons of body weight at specific time points

| **Group** | **Group Comparison** | **Mean Difference** | **Standard Error** | **Sequential Bonferroni *P* Value** |
| --- | --- | --- | --- | --- |
| **week1** | ST36 verum ACE **vs** Obese model control | -3.5773 | 1.6261 | 1 |
|  | ST36 verum **vs** ST36 sham ACE | -3.8523 | 1.02438 | 0.077 |
|  | ST36 verum **vs** ST25 ACE | -2.501 | 1.60192 | 1 |
|  | ST36 verum **vs** ST25+ ST36 ACE | -4.734 | 1.00495 | 0.001 |
| **week2** | ST36 verum ACE **vs** Obese model control | -3.0283 | 1.44072 | 1 |
|  | ST36 verum **vs** ST36 sham ACE | -2.4433 | 0.81666 | 1 |
|  | ST36 verum **vs** ST25 ACE | -1.649 | 1.35236 | 1 |
|  | ST36 verum **vs** ST25+ ST36 ACE | -3.401 | 0.90739 | 0.081 |
| **week5** | ST36 verum ACE **vs** Obese model control | -2.9163 | 1.3392 | 1 |
|  | ST36 verum **vs** ST36 sham ACE | -1.2519 | 0.92145 | 1 |
|  | ST36 verum **vs** ST25 ACE | -1.719 | 1.27811 | 1 |
|  | ST36 verum **vs** ST25+ ST36 ACE | -2.653 | 0.96846 | 1 |

**Abbreviations:** ST36, Zusanli; ST25, Tianshu; ACE, acupoint catgut embedding

## Supplementary Tables S5–S7：GEE results for Lee’s index

Table S5. GEE main effects for Lee’s index

| **Source** | **Wald *χ²*** | **Degrees of Freedom** | ***P* value** |
| --- | --- | --- | --- |
| **Group** | 27.268218 | 5 | ＜0.001 |
| **Time Points** | 612.982185 | 5 | ＜0.001 |
| **Group * Time Points** | 1434.034502 | 25 | ＜0.001 |

Table S6. Within-group comparisons of Lee’s index at different time points

| **Group** | **Time Point Comparison** | **Mean Difference** | **Standard Error** | **Sequential Bonferroni**  ***P* Value** |
| --- | --- | --- | --- | --- |
| Normal control  (*n*=6) | week0 vs week1 | -0.0004 | 0.00069 | 1 |
|  | week1 vs week5 | -0.0033 | 0.00138 | 1 |
| Obese model control  (*n*=6) | week0 vs week1 | 0.0168 | 0.00157 | ＜0.001 |
|  | week1 vs week5 | 0.0025 | 0.00108 | 1 |
| ST36 sham ACE  (*n*=9) | week0 vs week1 | 0.0147 | 0.00148 | ＜0.001 |
|  | week1 vs week5 | 0.0092 | 0.00271 | 0.307 |
| ST36 verum ACE  (*n*=10) | week0 vs week1 | 0.0235 | 0.00247 | ＜0.001 |
|  | week1 vs week5 | 0.0006 | 0.00216 | 1 |
| ST25 ACE  (*n*=10) | week0 vs week1 | 0.0154 | 0.00223 | ＜0.001 |
|  | week1 vs week5 | 0.003 | 0.0028 | 1 |
| ST25+ ST36 ACE  (*n*=10) | week0 vs week1 | 0.0148 | 0.00258 | ＜0.001 |
|  | week1 vs week5 | 0.0075 | 0.0019 | 0.034 |

**Abbreviations:** ST36, Zusanli; ST25, Tianshu; ACE, acupoint catgut embedding

Table S7. Between-group comparisons of Lee’s index at specific time points

| **Group** | **Group Comparison** | **Mean Difference** | **Standard Error** | **Sequential Bonferroni**  **P Value** |
| --- | --- | --- | --- | --- |
| **week1** | ST36 verum ACE **vs** Obese model control | -0.0046 | 0.0035 | 1 |
|  | ST36 verum **vs** ST36 sham ACE | -0.0035 | 0.00368 | 1 |
|  | ST36 verum **vs** ST25 ACE | -0.0097 | 0.00457 | 1 |
|  | ST36 verum **vs** ST25+ ST36 ACE | -0.0167 | 0.00302 | ＜0.001 |
| **week2** | ST36 verum ACE **vs** Obese model control | -0.0028 | 0.00305 | 1 |
|  | ST36 verum **vs** ST36 sham ACE | 0.0011 | 0.00316 | 1 |
|  | ST36 verum **vs** ST25 ACE | -0.007 | 0.00359 | 1 |
|  | ST36 verum **vs** ST25+ ST36 ACE | -0.0122 | 0.00266 | 0.002 |
| **week5** | ST36 verum ACE **vs** Obese model control | -0.0026 | 0.00322 | 1 |
|  | ST36 verum **vs** ST36 sham ACE | 0.0051 | 0.00337 | 1 |
|  | ST36 verum **vs** ST25 ACE | -0.0073 | 0.00312 | 1 |
|  | ST36 verum **vs** ST25+ ST36 ACE | -0.0098 | 0.00293 | 0.369 |

**Abbreviations:** ST36, Zusanli; ST25, Tianshu; ACE, acupoint catgut embedding

## Supplementary Tables S8-9: Cage-level weekly food intake data and linear mixed-model analysis

Supplementary Table S8. Cage-level weekly food intake during the intervention

| **Group** | **Treatment cage ID** | **Mice per cage** | **Food intake (g/mouse/week)** | | | | | |
| --- | --- | --- | --- | --- | --- | --- | --- | --- |
|  |  |  | **Baseline proxy** | **Week 1** | **Week 2** | **Week 3** | **Week 4** | **Week 5** |
| **Normal control** | 1 | 3 | 26.93 | 26.10 | 27.00 | 28.43 | 26.50 | 26.10 |
|  | 2 | 3 | 27.07 | 27.53 | 25.47 | 33.70 | 16.17 | 24.27 |
| **Obese model control** | 3 | 3 | 26.53 | 16.83 | 24.03 | 27.40 | 24.47 | 27.17 |
|  | 4 | 3 | 33.96 | 10.57 | 26.70 | 18.30 | 52.67 | 35.37 |
| **ST36 sham ACE** | 11 | 4 | 26.04 | 15.58 | 25.38 | 31.90 | 30.43 | 25.03 |
|  | 12 | 5 | 42.82 | 9.52 | 22.98 | 37.88 | 28.50 | 30.40 |
| **ST36 verum ACE** | 13 | 2 | 26.25 | 13.85 | 20.15 | 26.05 | 24.85 | 23.85 |
|  | 14 | 2 | 29.09 | 12.05 | 30.50 | 30.35 | 29.75 | 28.35 |
|  | 15 | 3 | 33.08 | 8.07 | 25.90 | 12.97 | 39.60 | 31.93 |
|  | 16 | 3 | 32.95 | 9.50 | 32.33 | 36.90 | 31.27 | 28.57 |
| **ST25 ACE** | 5 | 3 | 20.00 | 12.47 | 25.60 | 28.80 | 28.63 | 27.60 |
|  | 6 | 3 | 35.25 | 13.10 | 24.20 | 25.53 | 28.03 | 26.40 |
|  | 7 | 4 | 31.34 | 11.80 | 21.68 | 31.90 | 29.38 | 33.83 |
| **ST25+ST36 ACE** | 8 | 4 | 29.73 | 11.03 | 22.70 | 23.03 | 30.90 | 26.55 |
|  | 9 | 4 | 33.83 | 11.40 | 16.80 | 26.13 | 24.23 | 24.45 |
|  | 10 | 2 | 38.93 | 11.45 | 19.50 | 27.55 | 25.50 | 26.50 |

**Note:** Food intake is expressed as g/mouse/week. Each treatment cage was treated as the experimental unit, and baseline values are composition-weighted proxies.

Supplementary Table S9. Type III tests of fixed effects for cage-level changes in food intake

| **Effect** | **Numerator df** | **Denominator df** | ***F*** | ***P* value** |
| --- | --- | --- | --- | --- |
| **Group** | 5 | 10 | 1.160 | 0.392 |
| **Time** | 4 | 40 | 19.687 | <0.001 |
| **Group × Time** | 20 | 40 | 1.646 | 0.089 |

**Note:** A linear mixed-effects model was fitted using REML, with cage specified as the subject, week as the repeated

measure, and a compound-symmetry covariance structure.

## Supplementary Table S10. Leptin resistance–related molecular outcomes

| **Group** | **Serum Leptin(****ng/mL)** | **Cerebrospinal Leptin(ng/mL)** | **SOCS3** | **PTP1B** | **POMC mRNA** | **NPY mRNA** |
| --- | --- | --- | --- | --- | --- | --- |
| Normal control (*n*=6) | 4.4744(2.7166, 4.6994) *** | 4.7289±0.58544 | 0.8493(0.8257, 0.9395) | 0.7782±0.04333 | 1.0161±0.22034 | 1.0104±0.19512 |
| Obese model control(*n*=6) | 12.3377(11.787, 13.217) | 4.1489±0.64561 | 0.8285(0.7997, 0.9333) | 0.7413±0.0525 | 1.1594±0.23054 | 0.9438±0.12612 |
| ST36 sham ACE(*n*=9) | 8.0729(7.7135, 8.3736) | 5.9442±0.66265 | 0.6939(0.6503, 0.798) | 0.6387±0.0603△△△ | 1.6655±0.29641 | 0.6892±0.11906 |
| ST36 verum ACE(*n*=10) | 7.3198(6.4779, 9.3111) | 6.9417±0.81789*** | 0.5804(0.5187, 0.6069)** | 0.4592±0.05514*** | 2.0258±0.21388*** | 0.5807±0.09315*** |
| ST25 ACE  (*n*=10) | 9.2732(7.9343, 10.3633) | 5.664±1.46196 | 0.7306(0.694, 0.8239)# | 0.6622±0.05883### | 1.6144±0.25308# | 0.7759±0.14407# |
| ST25+ ST36 ACE(*n*=10) | 5.037(4.4875, 5.6448) | 7.3416±0.39176 | 0.5058(0.3957, 0.5582) | 0.3758±0.02731## | 2.1422±0.36108 | 0.5772±0.13711 |
| Statistical Value | *H*=41.180 | *F*=15.450 | *H*=39.972 | *F*=80.566 | *F*=20.404 | *F*=13.636 |
| *P* value | ＜0.001 | ＜0.001 | ＜0.001 | ＜0.001 | ＜0.001 | ＜0.001 |

**Symbol** * indicates comparison between the obese model control and experimental groups; △ indicates comparison between ST36 verum ACE and sham ACE groups; # indicates comparison between ST36 verum ACE and ST25 or ST36 + ST25 ACE groups.

**Significance:** * / △ / # for p<0.05; ** / △△ / ## for p<0.01; *** / △△△ / ### for p<0.001.

**Abbreviations:** ST36, Zusanli; ST25, Tianshu; ACE, acupoint catgut embedding

## Supplementary Table S11. Ghrelin–GOAT pathway–related molecular outcomes

| **Group** | **Gastric tissue Ghrelin(****ng/mL)** | **GOAT(ng/mL)** | **Cerebrospinal Ghrelin(ng/mL)** | **GHSR-1a****(pg/mL)** |
| --- | --- | --- | --- | --- |
| Normal control (*n*=6) | 8.6269±0.5691*** | 133.3642±4.7641*** | 8.4325(8.3604, 8.4956)*** | 897.8905(869.4626, 931.8971)*** |
| Obese model control (*n*=6) | 4.0879±0.40972 | 66.1844±13.24382 | 3.8678(3.7031, 3.9636) | 549.8719(500.9688, 584.5313) |
| ST36 sham ACE(*n*=9) | 5.892±0.79071△ | 97.9152±7.88451 | 5.5663(5.4243, 5.6237) | 618.894(584.828, 707.323) |
| ST36 verum ACE(*n*=10) | 6.8523±0.70987*** | 105.8021±7.63907*** | 7.3554(7.1966, 7.3867)** | 755.2171(694.9192, 805.8709)* |
| ST25 ACE (*n*=10) | 5.2225±0.66817### | 77.6341±7.47715### | 5.3015(5.2472, 5.4336) | 560.4952(469.811, 603.5524)## |
| ST25+ ST36 ACE(*n*=10) | 6.8227±0.69569 | 111.1201±11.14997 | 7.4534(7.3971, 7.541) | 763.5259(733.5846, 799.0458) |
| Statistical Value | *F*=35.595 | *F*=49.621 | *H*=43.915 | *H*=40.488 |
| *P* value | ＜0.001 | ＜0.001 | ＜0.001 | ＜0.001 |

Statistical symbols and are defined as in Table S8.
